# Mothers' Roles in Prevention and Care of Diarrhea in Children of Aran and Bidgol, Iran

**Published:** 2014-06-15

**Authors:** Farzaneh Saberi, Shima Amini, Razieh Jan Nesari

**Affiliations:** 1 Nursing and Midwifery Faculty, Kashan University of Medical Sciences, Kashan, IR Iran

**Keywords:** Knowledge, Diarrhea, Child

Dear Editor,

Diarrhea is the second leading cause of death in children under five in developing countries and one of the nine causes of death in children worldwide (1, 2). Diarrheal diseases are major causes of malnutrition, delayed physical development, and early childhood mortality in in developing countries and poor communities (3, 4). The major cause of death in children with diarrhea is loss of water and essential minerals. We can use the oral rehydration solution (ORS) to replace the lost minerals and water and prevent the progression of dehydration (1). Three main factors that can prevent children from death include educating the communities on the adequate fluid and nutrition intakes, continuing breastfeeding, and child care during diarrhea. Lack of knowledge in this area may lead to poor practices (4). According to various statistics about knowledge and practice of mothers in prevention and care of diarrheal diseases in children (4), this study was performed to assess this issue. This cross-sectional study was performed in fall 2013 on 81 mothers with children under five years having a history of diarrhea. They were selected from 117 mothers who had referred to health centers and rural health houses in Aran and Bidgol city, Iran, using convenience sampling. Data were collected with questionnaires which were completed by the interviewed mothers. The questionnaire consisted of five questions considering the demographic data, 26 questions related to the mothers' knowledge, and 14 questions related to the mothers' practices regarding prevention and care of children with diarrheal disease. Total score of knowledge was 26; 26-19 presented high, 18-10 average, and 9-0 low knowledge. Total score of practice was 14; 0-8 presented bad practice and 9-14 good practice. Data was analyzed by descriptive statistics, and chi-square test. Results showed that the mean age of mothers was 31.6 ± 2.7 years; 33.3% of mothers were graduated from high school; 37% were primipara and 63% were multipara. The knowledge level was moderate in 71.6% of mothers. Only 13.5% of the mothers had good knowledge. The mean score of mothers' knowledge about diarrhea and its causes was 13.9 ± 4.02. The mean score of mothers' practices was 8.5±1.8. Only about half of the mothers (53.1%) were aware of the role of ORS in dehydration treatment; 46.9% prepared ORS correctly and 34.6% were awarded for holding the period of ORS and acting accordingly. Of the mothers, 50.6% got good scores in practice and the rest got bad scores. None of the mothers got perfect knowledge and practice scores. However, with increased knowledge level, practice was better; but chi-square test did not show any significant relationship between the mothers´ knowledge (high, average, low) and practice (good, bad) regarding prevention and care of diarrheal diseases in children (P = 0.072). The results showed that the majority of mothers in the study had average levels of knowledge and practice. Due to serious complications of diarrhea, it is necessary to educate women about prevention and care of children with diarrhea.
